# Supplementary material for: Adverse pregnancy outcomes are associated with Plasmodium vivax malaria in a prospective cohort of women from the Brazilian Amazon
Source: PLoS Negl Trop Dis. 2021 Apr 29;15(4):e0009390. doi: 10.1371/journal.pntd.0009390 (PMC8112668; doi:10.1371/journal.pntd.0009390)
Supplement: S1 STROBE checklist — (DOCX) [file pntd.0009390.s001.docx]

STROBE Statement—Checklist of items that should be included in reports of ***cohort studies***

|  | Item No | Recommendation | Page | Relevant text from manuscript |
| --- | --- | --- | --- | --- |
| **Title and abstract** | 1 | (*a*) Indicate the study’s design with a commonly used term in the title or the abstract | 3 | Observational cohort study in the Brazilian Amazon between January 2013 and April 2015. |
|  |  | (*b*) Provide in the abstract an informative and balanced summary of what was done and what was found | 3 | See **Abstract** |
| Introduction | | |  |  |
| Background/rationale | 2 | Explain the scientific background and rationale for the investigation being reported | 7 | See **Introduction** |
| Objectives | 3 | State specific objectives, including any prespecified hypotheses | 7,8 | In this study, we aimed to evaluate the consequences of *P. vivax* infection during gestation on the health of mothers and their neonates. Herein, we report the deleterious relationship between *P. vivax* monoinfections, poor pregnancy outcomes, placental histopathology and, cytokine and angiogenic imbalance in pregnant women from the Brazilian Amazon. |
| Methods | | |  |  |
| Study design | 4 | Present key elements of study design early in the paper | 9 | See **Setting and study design** in Methods section. |
| Setting | 5 | Describe the setting, locations, and relevant dates, including periods of recruitment, exposure, follow-up, and data collection | 9-11 | See **Setting and study design, Malaria screening and treatment and Laboratory Procedures** in Methods section. |
| Participants | 6 | (*a*) Give the eligibility criteria, and the sources and methods of selection of participants | 9, 11 | See **Setting and study design** and **Exclusion criteria** in Methods section. |
| Variables | 7 | Clearly define all outcomes, exposures, predictors, potential confounders, and effect modifiers. Give diagnostic criteria, if applicable | 10-13 | See **Malaria screening and treatment**, **Laboratory procedures, Angiogenic factors and Leptin measurement, Cytokines/anaphylatoxins measurement by CBA, Measurement of total IgG antibodies against PvMSP1_19_, Placental weight and newborn anthropometric measurements,** and **Gestational age estimation and other definitions** in Methods section**.** |
| Data sources/ measurement | 8* | For each variable of interest, give sources of data and details of methods of assessment (measurement). Describe comparability of assessment methods if there is more than one group | 10-13 | See **Malaria screening and treatment**, **Laboratory procedures, Angiogenic factors and Leptin measurement, Cytokines/anaphylatoxins measurement by CBA, Measurement of total IgG antibodies against PvMSP1_19_, Placental weight and newborn anthropometric measurements,** and **Gestational age estimation and other definitions** in Methods section. |
| Bias | 9 | Describe any efforts to address potential sources of bias- | 9-11 | See **Setting and study design, Malaria screening and treatment, and Exclusion criteria** in Methods section.  The principal investigators from Department of Parasitology regularly visited the study sites and trained local staff on the case definition to minimize a selection bias due to different interpretations of the criteria. |
| Study size | 10 | Explain how the study size was arrived at | 9, 11 | See **Setting and study design and Exclusion criteria, and Figure 1** in Methods section. |
| Quantitative variables | 11 | Explain how quantitative variables were handled in the analyses. If applicable, describe which groupings were chosen and why | 14 | See **Statistical analysis.** |
| Statistical methods | 12 | (*a*) Describe all statistical methods, including those used to control for confounding | 14 | See **Statistical analysis.** |
|  |  | (*b*) Describe any methods used to examine subgroups and interactions | 14 | See **Statistical analysis.** |
|  |  | (*c*) Explain how missing data were addressed | - | N.A. |
|  |  | (*d*) If applicable, describe analytical methods taking account of sampling strategy | - | N.A. |
|  |  | (*e*) Describe any sensitivity analyses | - | N.A. |
| Results | | |  |  |
| Participants | 13* | (a) Report numbers of individuals at each stage of study—eg numbers potentially eligible, examined for eligibility, confirmed eligible, included in the study, completing follow-up, and analysed | 16-30 | See **Results.** |
|  |  | (b) Give reasons for non-participation at each stage | 10,11 | See **Figure 1** and **Exclusion criteria.** |
|  |  | (c) Consider use of a flow diagram | 10 | See **Figure 1.** |
| Descriptive data | 14* | (a) Give characteristics of study participants (eg demographic, clinical, social) and information on exposures and potential confounders | 9, 16- 26 | See **Setting and study design** and **Figure 1** in Methods section; **Study Population and Baseline Characteristics** - **Table 1 and Table 2, *P. vivax* Malaria in Pregnancy - Table 3** in Results section. |
|  |  | (b) Indicate number of participants with missing data for each variable of interest | 10, 17-26 | See **Figure 1**, and footnotes of each Table and Figure in **Results** section. |
| Outcome data | 15* | Report numbers of outcome events or summary measures | 19-30 | See **Table 2**, **Table 3**, **Figures 2-5** in Results section. |
| Main results | 16 | (*a*) Give unadjusted estimates and, if applicable, confounder-adjusted estimates and their precision (eg, 95% confidence interval). Make clear which confounders were adjusted for and why they were included | 14-30 | See **Statistical analysis** and **Results.** |
|  |  | (*b*) Report category boundaries when continuous variables were categorized | - | N.A. |
|  |  | (*c*) If relevant, consider translating estimates of relative risk into absolute risk for a meaningful time period | - | N.A. |
| Other analyses | 17 | Report other analyses done—eg analyses of subgroups and interactions, and sensitivity analyses | - | N.A. |
| Discussion | | |  |  |
| Key results | 18 | Summarise key results with reference to study objectives | 31-35 | See **Discussion.** |
| Limitations | 19 | Discuss limitations of the study, taking into account sources of potential bias or imprecision. Discuss both direction and magnitude of any potential bias | 31-35 | See **Discussion** |
| Interpretation | 20 | Give a cautious overall interpretation of results considering objectives, limitations, multiplicity of analyses, results from similar studies, and other relevant evidence | 31-35 | See **Discussion.** |
| Generalisability | 21 | Discuss the generalisability (external validity) of the study results | 31-35 | See **Discussion.** |
| Other information | | |  |  |
| Funding | 22 | Give the source of funding and the role of the funders for the present study and, if applicable, for the original study on which the present article is based | - | As required, it has been added to the Financial Disclosure section of the submission form. |

**Note:** *Give information separately for exposed and unexposed groups.

An Explanation and Elaboration article discusses each checklist item and gives methodological background and published examples of transparent reporting. The STROBE checklist is best used in conjunction with this article (freely available on the Web sites of PLoS Medicine at http://www.plosmedicine.org/, Annals of Internal Medicine at http://www.annals.org/, and Epidemiology at http://www.epidem.com/). Information on the STROBE Initiative is available at www.strobe-statement.org.
