# Supplementary material for: Adverse pregnancy outcomes are associated with Plasmodium vivax malaria in a prospective cohort of women from the Brazilian Amazon
Source: PLoS Negl Trop Dis. 2021 Apr 29;15(4):e0009390. doi: 10.1371/journal.pntd.0009390 (PMC8112668; doi:10.1371/journal.pntd.0009390)
Supplement: S1 Table — (DOCX) [file pntd.0009390.s002.docx]

**S1 Table. Characteristics of Anthropometric Measurements of Newborns, according to the gestational trimester in which the first infection occurred.**

| **Characteristics** | **Non-Infected**  **(N=169)** | ***P. vivax***  **(N=150)** | ***p*-value**^a^ | ***P. vivax* - 1^st^ tri**  **(N=46)** | ***p*-value**^b^ | ***P. vivax* - 2^nd^ tri**  **(N=54)** | ***p*-value**^c^ | ***P. vivax* - 3^rd^ tri**  **(N=50)** | ***p*-value**^d^ |
| --- | --- | --- | --- | --- | --- | --- | --- | --- | --- |
| Male newborns, n (%) | 77 (45.6) | 80 (53.3) | 0.18 | 25 (54.4) | 0.32 | 33 (61.1) | 0.06 | 22 (44.0) | 0.87 |
| Weight, g |  |  | 0.18 |  | 0.12 |  | 1.00 |  | 0.87 |
| Mean (SD) | 3229.4 ± 510.0 | 3151.0 ± 504.6 |  | 3064.9 ± 650.3 |  | 3211.4 ± 401.8 |  | 3165.1 ± 447.1 |  |
| Median (IQR) | 3255.0  (2975.0-3530.0) | 3132.5  (2840.0-3510.0) |  | 3060.0  (2695.0-3460.0) |  | 3230.0  (2900.0-3515.0) |  | 3072.5  (2835.0-3520.0) |  |
| Length, cm ^e^ |  |  | 0.67 |  | 0.33 |  | 0.96 |  | 0.99 |
| Mean (SD) | 49.0 ± 2.2 | 48.8 ± 2.2 |  | 48.4 ± 2.9 |  | 49.0 ± 1.9 |  | 49.0 ± 1.8 |  |
| Median (IQR) | 49.0 (48.0-50.0) | 49.0 (48.0-50.0) |  | 49.0 (47.0-50.0) |  | 49.0 (48.0-50.0) |  | 49.0 (48.0-50.0) |  |
| Rohrer index ^e, f^ |  |  | 0.11 |  | 0.28 |  | 0.99 |  | 0.44 |
| Mean (SD) | 2.7 ± 0.3 | 2.7 ± 0.3 |  | 2.7 ± 0.3 |  | 2.7 ± 0.3 |  | 2.7 ± 0.3 |  |
| Median (IQR) | 2.7 (2.6-2.9) | 2.7 (2.5-2.9) |  | 2.7 (2.4-2.8) |  | 2.7 (2.5-2.9) |  | 2.7 (2.5-2.9) |  |
| Head circumference, cm ^g^ |  |  | 0.05 |  | 0.007 |  | 1.00 |  | 0.68 |
| Mean (SD) | 34.2 ± 1.6 | 33.8 ± 1.6 |  | 33.4 ± 1.9 ^k^ |  | 34.2 ± 1.2 |  | 33.9 ± 1.6 |  |
| Median (IQR) | 34.0 (33.0-35.0) | 34.0 (33.0-35.0) |  | 34.0 (32.0-34.0) |  | 34.0 (34.0-35.0) |  | 34.0 (33.0-35.0) |  |
| Chest circumference, cm ^h^ |  |  | 0.05 |  | 0.006 |  | 0.93 |  | 0.99 |
| Mean (SD) | 33.7 ± 1.8 | 33.2 ± 2.3 |  | 32.6 ± 3.1 |  | 33.4 ± 1.9 |  | 33.6 ± 1.9 |  |
| Median (IQR) | 34.0 (33.0-35.0) | 33.0 (32.0-35.0) |  | 33.0 (32.0-34.0) |  | 33.0 (32.0-35.0) |  | 34.0 (33.0-35.0) |  |
| Apgar score 1 min ^i^ |  |  | 0.23 |  | 0.53 |  | 1.00 |  | 0.62 |
| Mean (SD) | 8.2 ± 1.4 | 8.4 ± 0.9 |  | 8.6 ± 0.6 |  | 8.2 ± 1.3 |  | 8.5 ± 0.6 |  |
| Median (IQR) | 9.0 (8.0-9.0) | 9.0 (8.0-9.0) |  | 9.0 (8.0-9.0) |  | 8.0 (8.0-9.0) |  | 9.0 (8.0-9.0) |  |
| Apgar score 5 min ^i^ |  |  | 0.14 |  | 0.60 |  | 0.96 |  | 0.50 |
| Mean (SD) | 9.3 ± 1.2 | 9.4 ± 0.6 |  | 9.5 ± 0.6 |  | 9.3 ± 0.6 |  | 9.5 ± 0.6 |  |
| Median (IQR) | 9.0 (9.0-10.0) | 9.0 (9.0-10.0) |  | 10.0 (9.0-10.0) |  | 9.0 (9.0-10.0) |  | 10.0 (9.0-10.0) |  |
| Fetal to placental weight ratio ^j^ |  |  | 0.58 |  | 0.43 |  | 0.99 |  | 0.90 |
| Mean (SD) | 5.7 ± 0.9 | 5.7 ± 0.9 |  | 5.9 ± 0.9 |  | 5.7 ± 0.8 |  | 5.6 ± 0.9 |  |
| Median (IQR) | 5.6 (5.1-6.0) | 5.7 (5.2-6.3) |  | 5.8 (5.3-6.4) |  | 5.7 (5.2-6.1) |  | 5.6 (5.1-6.2) |  |

Abbreviations: N, total number of individuals; tri, trimester; g, grams; cm, centimeters; SD, standard deviation; IQR, interquartile range. Statistical tests were applied according to the type of variable (Chi-square or Multiple linear regression, adjusted for maternal age, gravidity, residence, education, and occupation).

^a^ Differences between Non-Infected and *P. vivax* group.

^b^ Differences between Non-Infected and *P. vivax* infection in the 1^st^ trimester.

^c^ Differences between Non-Infected and *P. vivax* infection in the 2^nd^ trimester.

^d^ Differences between Non-Infected and *P. vivax* infection in the 3^rd^ trimester.

^e^ Length and Rohrer index were recorded in 166 non-infected and 148 *P. vivax* pregnant women.

^f^ The Rohrer index is the newborns’ weight in grams divided by the cube of the length in centimeters.

^g^ Head circumference was recorded in 167 non-infected and 148 *P. vivax* pregnant women.

^h^ Chest circumference was recorded in 160 non-infected and 130 *P. vivax* pregnant women.

^i^ Apgar score at 1 and 5 minutes were recorded in 164 non-infected and 131 *P. vivax* pregnant women.

^j^ Fetal to placental weight ratio was recorded in 155 non-infected and 126 *P. vivax* pregnant women. This ratio is the newborns’ weight divided by placental weight, both in grams.

^k^ Statistical difference for the comparison of *P. vivax* 1^st^ tri versus *P. vivax* 2^nd^ tri, *p* = 0.03.
