# Supplementary material for: Adverse pregnancy outcomes are associated with Plasmodium vivax malaria in a prospective cohort of women from the Brazilian Amazon
Source: PLoS Negl Trop Dis. 2021 Apr 29;15(4):e0009390. doi: 10.1371/journal.pntd.0009390 (PMC8112668; doi:10.1371/journal.pntd.0009390)
Supplement: S2 Table — (DOCX) [file pntd.0009390.s003.docx]

**S2 Table. Placental Parameters of Non-infected and *P. vivax*-infected women, according to the gestational trimester in which the first infection occurred.**

| **Characteristics** | **Non-Infected**  **N=155** | ***P. vivax***  **N=128** | ***p*-value**^a^ | ***P. vivax* - 1^st^ tri**  **(N=37)** | ***p*-value**^b^ | ***P. vivax* - 2^nd^ tri**  **(N=44)** | ***p*-value**^c^ | ***P. vivax* - 3^rd^ tri**  **(N=47)** | ***p*-value**^d^ |
| --- | --- | --- | --- | --- | --- | --- | --- | --- | --- |
| Placental histological parameters, median (IQR) |  |  |  |  |  |  |  |  |  |
| Syncytial nuclear aggregates | 13.0  (10.0-17.0) | 15.0  (11.0-21.0) | 0.001 | 19.0  (11.0-22.0) | 0.02 | 13.0  (10.0-18.0) | 0.90 | 16.0  (11.0-23.0) | 0.002 |
| Fibrin deposition score | 1.9  (1.9-2.8) | 2.5  (1.9-2.8) | 0.001 | 2.0  (1.9-2.8) | 0.22 | 2.4  (1.9-2.8) | 0.17 | 2.8  (2.0-2.8) | 0.01 |
| Fibrinoid necrosis ^e^ | 6.8  (3.9-10.2) | 7.5  (5.1-10.8) | 0.75 | 8.3  (6.2-12.6) | 0.47 | 6.7  (4.5-9.4) | 0.78 | 8.1  (5.1-11.1) | 1.00 |
| Placental barrier thickness ^f^ | 3.1  (2.8-3.5) | 3.1  (2.8-3.5) | 0.91 | 3.2  (2.8-3.6) | 0.95 | 3.1  (2.9-3.5) | 0.94 | 3.1  (2.6-3.4) | 0.49 |
| Villous vascularity ^g^ | 4.0  (3.5-4.4) | 4.0  (3.4-4.7) | 0.58 | 4.0  (3.5-4.4) | 1.00 | 4.5 ^k^  (3.6-5.3) | 0.15 | 3.9  (3.3-4.6) | 0.69 |
| Proliferation index ^h^ | 3.5  (2.6-4.7) | 3.7  (2.6-4.7) | 0.95 | 3.7  (3.0-4.9) | 1.00 | 3.6  (2.6-4.6) | 0.99 | 3.9  (2.5-4.7) | 1.00 |
| Leukocytes infiltrate ^i^ | 15.0  (9.0-21.0) | 21.5  (14.0-29.0) | 0.004 | 21.0  (14.0-28.0) | 0.09 | 21.0  (14.0-29.0) | 0.16 | 23.0  (14.0-30.0) | 0.29 |
| Monocytes/macrophages ^j^ | 4.0  (2.0-6.0) | 8.0  (6.0-13.0) | < 0.0001 | 7.0  (5.0-9.5) | 0.01 | 8.5  (5.5-13.0) | 0.0001 | 9.0  (6.0-15.0) | < 0.0001 |

Abbreviations: N, total number of individuals; tri, trimester. Results are presented as median and interquartile range (IQR). Differences between groups were determined by Multiple linear regression, adjusted for maternal age, gravidity, residence, education, and occupation.

^a^ Differences between Non-Infected and *P. vivax* group.

^b^ Differences between Non-Infected and *P. vivax* infection in the 1^st^ trimester.

^c^ Differences between Non-Infected and *P. vivax* infection in the 2^nd^ trimester.

^d^ Differences between Non-Infected and *P. vivax* infection in the 3^rd^ trimester.

^e^ Fibrinoid necrosis was recorded in placentas from 121 *P. vivax* pregnant women.

^f^ Placental barrier thickness was recorded in placentas from 127 *P. vivax* pregnant women.

^g^ Villous vascularity was recorded in placentas from 151 non-infected and 115 *P. vivax* pregnant women.

^h^ Proliferation index was recorded in placentas from 153 non-infected and 126 *P. vivax* pregnant women.

^i^ Leukocyte infiltrate (CD45+) was recorded in placentas from 153 non-infected and 118 *P. vivax* pregnant women.

^j^ Monocytes/macrophages infiltrate (CD68+) was recorded in placentas from 154 non-infected and 127 *P. vivax* pregnant women.

^k^ Statistical difference for the comparison of *P. vivax* 2^nd^ tri versus *P. vivax* 3^rd^ tri, *p* = 0.04.
