# Supplementary material for: Adverse pregnancy outcomes are associated with Plasmodium vivax malaria in a prospective cohort of women from the Brazilian Amazon
Source: PLoS Negl Trop Dis. 2021 Apr 29;15(4):e0009390. doi: 10.1371/journal.pntd.0009390 (PMC8112668; doi:10.1371/journal.pntd.0009390)
Supplement: S3 Table — (DOCX) [file pntd.0009390.s004.docx]

**S3 Table. Angiogenic factors in placental plasma from Non-infected and** ***P. vivax*-infected women,** **according to the gestational trimester in which the first infection occurred.**

| **Characteristics** | **Non-Infected**  **(N=151)** | ***P. vivax***  **(N=120)** | ***p*-value**^a^ | ***P. vivax* - 1^st^ tri**  **(N=37)** | ***p*-value**^b^ | ***P. vivax* - 2^nd^ tri**  **(N=41)** | ***p*-value**^c^ | ***P. vivax* - 3^rd^ tri**  **(N=42)** | ***p*-value**^d^ |
| --- | --- | --- | --- | --- | --- | --- | --- | --- | --- |
| Angiogenic factors, median (IQR) ^e^ |  |  |  |  |  |  |  |  |  |
| ANG-1 | 15.7  (10.2-22.9) | 16.6  (11.0-25.3) | 0.16 | 15.7  (11.3-20.7) | 1.00 | 17.0  (13.2-26.6) | 0.18 | 16.1  (10.2-25.7) | 0.80 |
| ANG-2 ^f^ | 6.8  (3.5-12.4) | 4.0  (1.2-7.7) | < 0.0001 | 4.4  (1.0-6.6) | 0.03 | 4.0  (1.3-7.1) | 0.12 | 3.8  (1.4-10.1) | 0.05 |
| ANG-2/ANG-1 ratio | 0.4  (0.2-1.0) | 0.2  (0.1-0.6) | 0.09 | 0.2  (0.1-0.6) | 0.58 | 0.2  (0-0.6) | 0.26 | 0.3  (0.1-0.6) | 0.95 |
| sTIE-2 ^f^ | 13.9  (9.1-19.0) | 17.3  (11.7-22.0) | 0.006 | 15.7  (12.6-19.2) | 0.91 | 17.2  (10.9-23.2) | 0.12 | 18.5  (11.7-24.7) | 0.008 |
| sTIE-2/ANG-1 ratio | 0.8  (0.6-1.2) | 1.0  (0.7-1.4) | 0.87 | 1.0  (0.8-1.2) | 1.00 | 0.9  (0.6-1.1) | 0.85 | 1.0  (0.7-1.5) | 0.52 |
| VEGF-A | 3.2  (1.8-4.7) | 4.0  (2.1-5.2) | 0.67 | 4.0  (2.1-5.1) | 0.97 | 4.2  (2.0-5.3) | 0.98 | 3.5  (2.2-5.0) | 1.00 |
| sFlt-1 ^f^ | 80.4  (35.4-121.5) | 55.5  (7.3-123.1) | 0.02 | 62.6  (17.8-125.6) | 0.51 | 62.6  (5.7-122.4) | 0.28 | 48.9  (6.0-122.7) | 0.29 |
| sVEGFR-2 ^f^ | 14.4  (12.8-15.8) | 14.9  (13.3-16.3) | 0.009 | 14.8  (13.2-16.4) | 0.57 | 14.9  (13.8-16.7) | 0.02 | 14.9  (13.2-15.8) | 0.46 |
| Leptin ^g^ | 29.7  (16.5-46.0) | 20.2  (10.5-37.5) | 0.04 | 20.2  (11.0-43.6) | 0.44 | 22.6  (9.3-35.7) | 0.50 | 18.3  (10.7-30.5) | 0.47 |

Abbreviations: N, total number of individuals; tri, trimester. Results are presented as median and interquartile range (IQR). Differences between groups were determined by Multiple linear regression, adjusted for maternal age, gravidity, residence, education, and occupation.

^a^ Differences between Non-Infected and *P. vivax* group.

^b^ Differences between Non-Infected and *P. vivax* infection in the 1^st^ trimester.

^c^ Differences between Non-Infected and *P. vivax* infection in the 2^nd^ trimester.

^d^ Differences between Non-Infected and *P. vivax* infection in the 3^rd^ trimester.

^e^ Angiogenic factors: ANG-1 and 2 angiopoietin-1 and 2, sTIE-2 soluble TEK receptor tyrosine kinase, VEGF-A vascular endothelial growth factor A, sFlt-1 fms-like tyrosine kinase-1 and sVEGFR-2 soluble vascular endothelial growth factor receptor 2. Shown in ng/mL.

^f^ ANG-2, sTIE-2, sFlt-1 and sVEGFR-2 were recorded in placental plasma from 150 non-infected pregnant women.

^g^ Leptin was recorded in placental plasma from 150 non-infected, 119 *P. vivax* group pregnant women. Shown in ng/mL.
