# Supplementary material for: Adverse pregnancy outcomes are associated with Plasmodium vivax malaria in a prospective cohort of women from the Brazilian Amazon
Source: PLoS Negl Trop Dis. 2021 Apr 29;15(4):e0009390. doi: 10.1371/journal.pntd.0009390 (PMC8112668; doi:10.1371/journal.pntd.0009390)
Supplement: S4 Table — (DOCX) [file pntd.0009390.s005.docx]

**S4 Table.** **Inflammatory factors in placental plasma from Non-infected and *P. vivax*-infected women, according to the gestational trimester in which the first infection occurred.**

| **Characteristics** | **Non-Infected**  **(N=151)** | ***P. vivax***  **(N=120)** | ***p*-value**^a^ | ***P. vivax* - 1^st^ tri**  **(N=37)** | ***p*-value**^b^ | ***P. vivax* - 2^nd^ tri**  **(N=41)** | ***p*-value**^c^ | ***P. vivax* - 3^rd^ tri**  **(N=42)** | ***p*-value**^d^ |
| --- | --- | --- | --- | --- | --- | --- | --- | --- | --- |
| Cytokines, pg/mL, median (IQR) ^e^ |  |  |  |  |  |  |  |  |  |
| IL1-β | 4.7  (3.9-5.9) | 4.4  (3.5-5.7) | 0.36 | 4.2  (3.8-6.0) | 0.91 | 3.9 ^g^  (3.4-5.5) | 0.83 | 4.7  (3.8-6.1) | 0.10 |
| IL-6 | 75.8  (34.3-135.8) | 47.4  (22.5-123.5) | 0.45 | 50.7  (21.5-187.2) | 0.12 | 40.5  (23.7-116.8) | 0.96 | 54.1  (22.6-116.8) | 0.99 |
| IL-8 | 25.7  (14.7-52.1) | 23.9  (14.7-52.9) | 0.40 | 26.4  (14.7-79.4) | 0.79 | 24.3  (14.3-44.4) | 0.97 | 21.9  (15.0-51.1) | 0.53 |
| IL-10 | 3.9  (3.1-4.9) | 4.1  (3.1-5.3) | 0.14 | 3.9  (3.3-5.3) | 0.10 | 3.9  (2.8-5.2) | 1.00 | 4.2  (3.3-5.5) | 0.90 |
| IL-12 | 3.6  (3.0-4.2) | 3.5  (2.8-4.1) | 0.01 | 3.3  (2.6-3.8) | 0.009 | 3.6  (2.7-3.9) | 0.19 | 3.7  (3.0-4.4) | 1.00 |
| TNF-α | 5.3  (4.2-6.1) | 5.0  (4.0-5.8) | 0.69 | 4.6 ^h^  (3.7-5.3) | 0.46 | 4.8  (4.0-5.6) | 0.71 | 5.4  (4.2-6.3) | 0.23 |
| Anaphylatoxins, pg/mL, median (IQR) ^f^ |  |  |  |  |  |  |  |  |  |
| C3a | 4.5  (3.3-6.5) | 5.4  (3.8-8.0) | 0.30 | 6.3  (4.0-8.0) | 0.84 | 5.4  (4.3-8.0) | 0.92 | 4.4  (3.2-7.3) | 0.93 |
| C4a | 34.1  (21.9-52.5) | 32.2  (19.2-56.2) | 0.22 | 34.9  (20.5-76.0) | 0.68 | 31.7  (26.9-54.8) | 0.98 | 32.6  (12.9-56.2) | 0.78 |
| C5a | 1117.2  (814.8-1475.5) | 834.6  (534.9-1005.6) | < 0.0001 | 807.1  (534.9-913.9) | 0.03 | 986.6  (699.2-1301.8) | 0.39 | 673.0  (445.9-871.3) | 0.006 |

Abbreviations: N, total number of individuals; tri, trimester. Results are presented as median and interquartile range. Differences between groups were determined by Multiple linear regression, adjusted for maternal age, gravidity, residence, education, and occupation.

^a^ Differences between Non-Infected and *P. vivax* group.

^b^ Differences between Non-Infected and *P. vivax* infection in the 1^st^ trimester.

^c^ Differences between Non-Infected and *P. vivax* infection in the 2^nd^ trimester.

^d^ Differences between Non-Infected and *P. vivax* infection in the 3^rd^ trimester.

^e^ Cytokines: IL-1β denotes interleukin-1 beta, IL6 interleukin-6, IL8 interleukin-8, IL10 interleukin-10, IL12 interleukin-12, and TNF-α tumor necrosis factor α. Shown in pg/mL.

^f^ C3a, C4a and C5a were recorded in placental plasma from 145 non-infected and 58 *P. vivax*-infected pregnant women.

^g^ Statistical difference for the comparison of *P. vivax* 2^nd^ tri versus *P. vivax* 3^rd^ tri, *p* = 0.04.

^h^ Statistical difference for the comparison of *P. vivax* 1^st^ tri versus *P. vivax* 3^rd^ tri, *p* = 0.03.
