# Supplementary material for: Adverse pregnancy outcomes are associated with Plasmodium vivax malaria in a prospective cohort of women from the Brazilian Amazon
Source: PLoS Negl Trop Dis. 2021 Apr 29;15(4):e0009390. doi: 10.1371/journal.pntd.0009390 (PMC8112668; doi:10.1371/journal.pntd.0009390)
Supplement: S5 Table — (DOCX) [file pntd.0009390.s006.docx]

**S5 Table. Anthropometric characteristics of the newborns, according to maternal previous exposure to *P. vivax* (PvMSP1).**

| **Characteristics** | **PvMSP1 Ab-**  **(N=112)** | **PvMSP1 Ab+**  **(N=58)** | ***p*-value^a^** | **PvMSP1 Ab-**  **(N=19)** | **PvMSP1 Ab+**  **(N=95)** | ***p*-value^b^** |
| --- | --- | --- | --- | --- | --- | --- |
|  | **Non-infected** | |  | ***Pv*-infected** | |  |
| IgG total (RI), median (IQR) ^c^ | 0 (0-0.3) | 4.0 (2.1-9.8) ^h^ | 0.10 | 0 (0-0.2) | 65.1 (14.1-91.6) | < 0.0001 |
| Newborn weight, Kg, median (IQR) ^d^ | 3225.0 (2940.0-3450.0) | 3370.0 (3015.0-3675.0) | 0.88 | 2850.0 (2600.0-3295.0) | 3142.0 (2867.5-3500.0) | 0.54 |
| Head circumference, cm, median (IQR) ^e^ | 34.0 (33.0-35.0) | 34.0 (34.0-35.0) | 0.78 | 33.0 (32.0-35.0) | 34.0 (33.0-35.0) | 0.82 |
| Chest circumference, cm, median (IQR) ^f^ | 34.0 (33.0-35.0) | 34.0 (33.0-35.0) | 0.84 | 33.5 (31.0-34.0) | 33.0 (32.0-34.0) | 0.88 |
| Length, cm, median (IQR) ^g^ | 49.0 (48.0-50.0) | 50.0 (49.0-50.0) | 0.45 | 49.0 (48.0-49.0) | 49.0 (48.0-50.0) | 0.90 |

Abbreviations: N, total number of individuals; RI, reactivity indices; Kg, kilograms; cm, centimeters. Results are presented as median and interquartile range (IQR). Differences between each group were determined by Multiple linear regression, adjusted for maternal age, gravidity, residence, education, and occupation.

^a^ Comparisons between Non-Infected groups.

^b^ Comparisons between *P. vivax*-infected groups.

^c^ Test using peripheral blood samples collected at recruitment.

^d^ Newborn weight was recorded in 111 non-infected Ab-, 58 non-infected Ab+, and 88 *P. vivax* Ab+.

^e^ Head circumference was recorded in 110 non-infected Ab-, 57 non-infected Ab+, and 87 *P. vivax* Ab+.

^f^ Chest circumference was recorded in 106 non-infected Ab-, 54 non-infected Ab+, 12 *P. vivax* Ab-, and 80 *P. vivax* Ab+.

^g^ Length was recorded in 109 non-infected Ab-, 57 non-infected Ab+, and 87 *P. vivax* Ab+.

^h^ Statistical difference for the comparison of non-infected Ab+ versus *P. vivax* Ab+, *p* < 0.0001.
