# Supplementary material for: Adverse pregnancy outcomes are associated with Plasmodium vivax malaria in a prospective cohort of women from the Brazilian Amazon
Source: PLoS Negl Trop Dis. 2021 Apr 29;15(4):e0009390. doi: 10.1371/journal.pntd.0009390 (PMC8112668; doi:10.1371/journal.pntd.0009390)
Supplement: S6 Table — (DOCX) [file pntd.0009390.s007.docx]

**S6 Table. Association between adverse pregnancy outcomes and *P. vivax* infections according to gravidity.**

| **Outcomes** | **aOdds Ratio**  **(95% CI)** | ***p*-value** |
| --- | --- | --- |
| **Preterm birth** |  |  |
| Multi | 1 |  |
| Primi | 1.72 (0.56-5.27) | 0.34 |
| **Low birth weight** |  |  |
| Multi | 1 |  |
| Primi | 2.54 (0.76-8.49) | 0.13 |
| **Term low birth weight** |  |  |
| Multi | 1 |  |
| Primi | 3.01 (0.60-15.48) | 0.18 |
| **Small for gestational age** |  |  |
| Multi | 1 |  |
| Primi | 0.70 (0.20-2.42) | 0.58 |
| **Reduced head circumference** |  |  |
| Multi | 1 |  |
| Primi | 2.64 (0.87-8.06) | 0.09 |
| **Reduced length** |  |  |
| Multi | 1 |  |
| Primi | 1.73 (0.58-5.12) | 0.32 |

Abbreviations: Multi, multigravidae; Primi, primigravidae; aOdds Ratio, adjusted Odds Ratio; CI, confidence interval. Odds ratios were adjusted (aOR) for maternal age, residence, education level and occupation. Preterm birth – birth < 37^th^ week of gestation; Low birth weight – birth weight < 2500 g; Term low birth weight – birth weight < 2500 g from 37^th^ week of gestation; Small for gestational age, Reduced head circumference and Reduced body length – birth weight, head circumference and length (respectively) < 10^th^ centile for sex-specific gestational age. *P-values* were estimated through multiple logistic regression methods.
