# Supplementary material for: Adverse pregnancy outcomes are associated with Plasmodium vivax malaria in a prospective cohort of women from the Brazilian Amazon
Source: PLoS Negl Trop Dis. 2021 Apr 29;15(4):e0009390. doi: 10.1371/journal.pntd.0009390 (PMC8112668; doi:10.1371/journal.pntd.0009390)
Supplement: S1 Text — (DOCX) [file pntd.0009390.s008.docx]

| S1 Text | Analysis Plan |
| --- | --- |

**Adverse pregnancy outcomes are associated with *P. vivax* malaria in a prospective cohort of women from the Brazilian Amazon**

**Principal Researchers**

Jamille Gregório Dombrowski

Rodrigo Medeiros de Souza

Sabrina Epiphanio

Lígia Antunes Gonçalves

Cláudio Romero Farias Marinho

This document was written in order to summarize the study protocol, and is in accordance with the project submitted to the Research Ethics Committee of the University of São Paulo in October 2012.

Protocol Version: 2.0

**Original project title:** Association between gestational malaria, intrauterine growth restriction and low birth weight in the Far-Western Brazilian Amazon

**Background**

Malaria is a major public health problem in different regions of the world, especially in developing countries, causing one million deaths annually, mainly children and pregnant women [1]. Malaria in pregnancy is characterized by the presence of *Plasmodium* spp. in the peripheral blood or the placenta [2], and may result in maternal anemia, intrauterine growth restriction (IUGR), low birth weight (LBW) and reduced fetal viability [3]. *Plasmodium vivax* (*P. vivax*) is the dominant species of malaria outside Africa, accounting for 25-40% of malaria cases worldwide [4]. In Brazil, where the prevalence of infections by *P. vivax* is also observed, about 95% of cases occur in the Amazon region, where nearly 500,000 new cases are reported every year [5]. Despite the high prevalence and possible association with major complications during pregnancy, the effects of infection by *P. vivax* in pregnant women are still not fully understood. Both IUGR and LBW are strongly associated with neonatal mortality and morbidity, as well as inhibited growth and cognitive development in children with chronic diseases in adulthood [6]. There are few studies on the effects of the disease on pregnant women and their fetuses, despite the high concentration of malaria cases in the Amazon region.

**Aims and objectives**

This project aims to investigate the association of gestational malaria with newborns anthropometric alterations in an malaria endemic area. Therefore, by analyzing different parameters, it is intended to:

i) Assess the incidence of *P. vivax*, *P. falciparum* and mixed infection (*P. falciparum* + *P. vivax*) in peripheral blood, umbilical cord, and placenta, comparing the frequency of miscarriages/abortion, stillbirth, LBW, and premature delivery in these groups.

ii) Study the consequences and the extent of the effect of malaria during pregnancy in the newborns, identifying qualitatively and quantitatively the correlation of the intensity of placental lesions with altered newborns’ anthropometric measures, and poor pregnancy outcomes.

**Methods**

*Types of studies*

Prospective cohort study.

*Types of participants*

Non-infected and *Plasmodium*-infected pregnant women enrolled through volunteer sampling before pregnancy outcome is known.

*Setting*

The cohort study will be conducted in the Amazonian region of the “Alto do Juruá” valley (Acre, Brazil), evaluating maternal-child pairs data of births that occurred at the general maternity, Hospital da Mulher e da Criança do Juruá (HMCJ, Cruzeiro do Sul), where approximately 90% of the total deliveries in the region occur. “Alto do Juruá” valley has high malaria endemicity.

*Study overview*

For each pregnant woman with malaria, two pregnant women with no history for this disease during the current pregnancy will be included, selected during prenatal care in the main Health Centers of Juruá region (Table 1). At recruitment, socioeconomic and clinical/obstetric data, peripheral blood, and a thick and thin blood smear to diagnose malaria will be collected. The pregnant women will be followed by a trained nurse, with at least two domiciliary visits to monitor their clinical state and collect peripheral blood samples. Whenever an episode of malaria occurs, an additional visit and blood sample will be collected. At delivery, clinical data from the mother and the newborn will be collected, as well biological samples from placenta and blood (Fig 1 and Table 2). All the women who develop malaria during pregnancy will be treated with antimalarial drugs under medical prescription, according to the Brazilian Ministry of Health guidelines.

**Table 1. Cohort groups by infection status.**

| ID Group | Number of individuals | Interventions to be performed |
| --- | --- | --- |
| *P. vivax* Group | 200 | Morbidity surveillance by clinical examination and questionnaire application. Fortnight and month peripheral blood collections for therapeutic control. |
| *P. falciparum* Group | 200 | Morbidity surveillance by clinical examination, questionnaire application. Fortnightly and monthly peripheral blood collections for healing control. |
| Non-Infected Group | 400 | Follow-up by clinical examination and questionnaire application. |


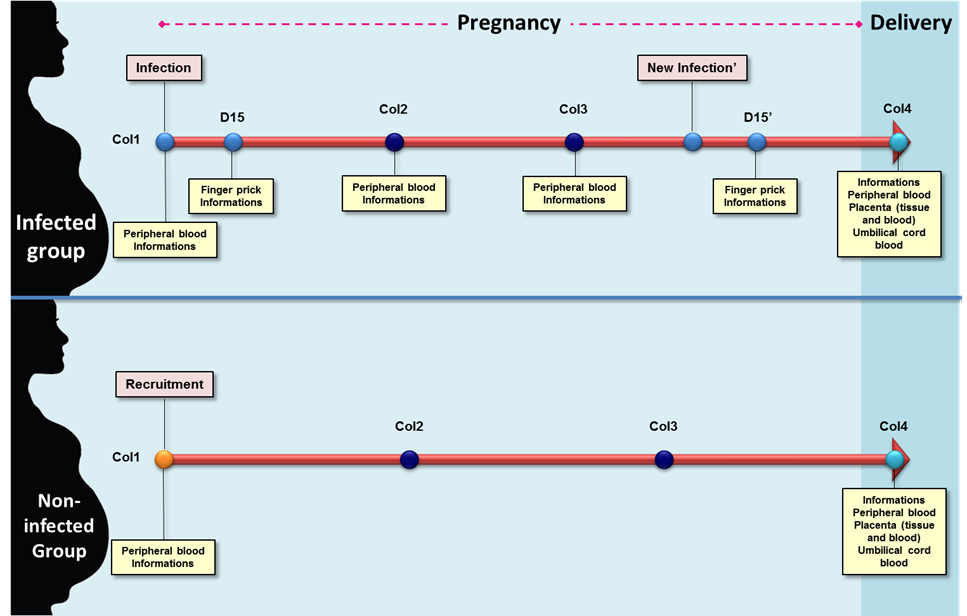


**Fig 1.** **Prospective cohort study time-line.** Col – collection; D – day after infection.

**Table 2. Maternal visits and procedures schedule.**

| Study Procedures | Visit 1  Enrollment | Visit 2 | Visit 3 | Visit 4  Malaria episode* | Visit 5  Cure check* | Visit 6  Delivery |
| --- | --- | --- | --- | --- | --- | --- |
| Written informed consent | X |  |  |  |  |  |
| Demographics/Clinical history | X |  |  |  |  |  |
| Physical examination / Clinical | X | X | X |  |  |  |
| Peripheral venous blood (mother) | X | X | X | X |  | X |
| Blood smear | X | X | X | X | X | X |
| Evaluation of morbidity |  | X | X |  |  |  |
| Malaria information |  |  |  | X |  |  |
| Treatment verification |  |  |  |  | X |  |
| Placental biopsy |  |  |  |  |  | X |
| Placental blood sample |  |  |  |  |  | X |
| Umbilical cord blood sample |  |  |  |  |  | X |
| Newborn anthropometric measures |  |  |  |  |  | X |

* Protocol followed for all episodes of malaria throughout pregnancy.

*Newborn anthropometric measures*

The newborn anthropometric measures will be obtained immediately after the delivery, maximum within 24h, by trained nurses. Weight will be measured in grams using digital pediatric scales, and the length and occipitofrontal head circumference will be measured in centimeters, using a non-stretching flexible measuring tape following the standards of the World Health Organization and Brazilian Ministry of Health.

**Outcomes**

Outcomes of interests encompassed the following adverse pregnancy outcomes:

- Incidence of clinical malaria during pregnancy
- Prevalence of *Plasmodium* parasitemia during pregnancy (blood smear and molecular diagnostic)
- Prevalence of *Plasmodium* parasitemia at delivery (blood smear and molecular diagnostic)
- Prevalence of placental *Plasmodium* infection (histology, blood smear, and molecular diagnostic)
- Miscarriage/Abortion: defined as a confirmed pregnancy that fails to progress, resulting in death and expulsion of the embryo or unviable fetus (< 22 weeks). Miscarriages will be analyzed as a dichotomous variable either present or absent.
- Stillbirth: defined as a baby born with no signs of life at or after 22 weeks' gestation.
- Preterm delivery: defined as babies born alive before 37 weeks of pregnancy are completed.
- Low birth weight: defined as a weight of less than 2500 g (up to and including 2499 g) irrespective of the gestational age.
- Small for gestational age (SGA), reduced head circumference, and reduced length were defined as: birth weight, head circumference, and length below the 10th centile sex-specific for gestational age.

**Risks**

Formation of hematoma at the site of venipuncture and discomfort at the fingertip after peripheral blood collection.

**Benefits**

Pregnant women will have clinical monitoring throughout the study. Furthermore, clinical malaria diagnosis will be carried out in a more specific manner. Pregnant women will also receive guidance for the treatment of gestational malaria. Finally, there will be a contribution to greater epidemiological surveillance.

**Eligibility criteria**

This study will include pregnant women, regardless of age, presenting abortion, vaginal delivery, or cesarean section of any gravity with any gestational age who have or not, in the gestation period, infections by *Plasmodium sp*. when accepting participating in the study. Their children will also be included in the study. Smokers, hypertensive, who had pre-eclampsia, eclampsia, hemorrhage in the 2^nd^ and 3^rd^ quarters, diabetes, multiple pregnancies, and other infections will be excluded from analysis.

**Confidentiality and data handling**

All data will be treated with confidentiality according to Good Clinical Practice. All raw data will be stored on a secure server in a password protected database.

**Data Analysis**

Data will be analyzed using R (r-project.org), Stata (StataCorp) and GraphPad Prism software. During analysis, means and standard deviations for normal quantitative variables, frequency distribution and proportions for qualitative variables will be calculated. Variables will also be described by measures of central tendency, dispersion, confidence intervals and medians for non-normal distribution. The Mantel Haenszel chi-square test with Yates' correction or Fisher's exact test will be used for proportions, and the Student t test for comparison or to establish statistically significant differences between means and the Mann-Whitney test will be used as a non-parametric alternative to the t test. Statistical significance level lower than or equal to 5% (*p* < 0.05) will be considered to reject the null hypothesis at 95% confidence interval (95% CI). To establish an association between primary and secondary variables, the odds ratio (OR) will be estimated using the same 95% CI and *p* < 0.05. Associations between lesions and other factors and covariates will be explored with standard techniques such as regression and correlation analysis and analysis of variance (ANOVA) with the aim of controlling confounding factors.

**Ethical considerations**

Ethical clearance was provided by the committees for research of the University of São Paulo and the Federal University of Acre (Plataforma Brasil, CAAE: 03930812.8.0000.5467 and 03930812.8.3001.5010, respectively), according to Resolution nº 196/96 of Brazilian National Health Committee. All the study participants or their legal guardians (if minors) gave written informed consent. The authors have agreed to maintain the confidentiality of the data collected from the medical records and databases, by signing the Term of Commitment for the Use of Data from Medical Records. The study was conducted in accordance with the Declaration of Helsinki and is registered in the Brazilian Clinical Trials Registry as RBR-3yrqfq.

**References**

1. World Health Organization. World Malaria Report 2011. 2011.

2. Brabin BJ. An analysis of malaria in pregnancy in Africa. Bull World Health Organ. 1983;61: 1005–16. Available: http://www.pubmedcentral.nih.gov/articlerender.fcgi?artid=2536236&tool=pmcentrez&rendertype=abstract

3. Chagas ECDS, Nascimento CT Do, Santana Filho FS De, Bôtto-Menezes CH, Martinez-Espinosa FE. Malária durante a gravidez: efeito sobre o curso da gestação na região amazônica. Rev Panam Salud Pública. 2009;26: 203–208. doi:10.1590/S1020-49892009000900003

4. Nosten F, McGready R, Simpson JA, Thwai KL, Balkan S, Cho T, et al. Effects of Plasmodium vivax malaria in pregnancy. Lancet. 1999;354: 546–549. doi:10.1016/S0140-6736(98)09247-2

5. BRASIL. Ministério da Saúde. Secretaria de Vigilância em Saúde. Situação epidemiológica da malária no Brasil, 2000 a 2011. 2013;44: 1–16.

6. United Nations Children’s Fund and World Health Organization. Low Birthweight: Country, regional and global estimates. 2004.
