# Supplementary material for: Adverse pregnancy outcomes are associated with Plasmodium vivax malaria in a prospective cohort of women from the Brazilian Amazon
Source: PLoS Negl Trop Dis. 2021 Apr 29;15(4):e0009390. doi: 10.1371/journal.pntd.0009390 (PMC8112668; doi:10.1371/journal.pntd.0009390)
Supplement: Attachment — Submitted filename: REPLY_REVIEWERS_COMMENTS_FINAL.docx [file pntd.0009390.s010.docx]

**Reviewer #1**

The authors have studied the impact of P. vivax infection on pregnancy outcome in a large prospective cohort of Brazilian women. Maternal history of infection during the pregnancy, placental histopathology, levels of angiogenic factors and cytokines, and levels of PvMSP1-19 antibodies are related to various measures of pregnancy outcome. The authors conclude that P. vivax infection, particularly during the first trimester, negatively affects pregnancy outcome.

**Methods**

*1) The authors use the terms malaria in pregnancy and placental malaria more or less randomly. In my opinion, placental malaria should be reserved for infections, where a placental disease focus is known or highly likely. Whereas this is generally the case for P. falciparum infection among women living in areas with stable parasite transmission, it remains highly controversial whether selective placental accumulation of P. vivax-infected erythrocytes occurs. As the authors do not provide such evidence, I strongly recommend that the authors use the term malaria in pregnancy (or pregnancy-associated malaria) throughout. This recommendation is supported by the fact that the authors did not find a statistically significant relation between their outcome variables and parity. Such a relation would be strongly expected in the case of bona fide placental malaria.*

R: We agree with this view and have now changed the term “*placental malaria*” to “*malaria in pregnancy*” in the appropriate places.

*2) The study appears well designed and it is nicely presented. My overriding concern is whether the uninfected and infected women can be compared directly, as they are throughout the manuscript. The reason for this concern is Table 1, where it is stated that 44/159 infected women lived in a rural area, whereas only 8/170 of the non-infected did. This difference is very highly significant – but not commented on in the manuscript. It is therefore an open – and critical – question whether it is justified to attribute the differences between the infected and uninfected women (only) to P. vivax infection. While it appears reasonable to suspect that a strongly pro-inflammatory infection such as P. vivax malaria would negatively affect pregnancy, it is also very highly likely that rural residence for many reasons other than P. vivax exposure predisposes for a pro-inflammatory status. The authors should comment on this, and definitely control for this likely confounding factor in their analyses wherever possible (i.e., a multivariate approach). Additional factors reported in Table 1 that might bias the data in the same direction (albeit less pronounced manner) are age, occupation, and educational level.*

R: We thank the reviewer for pointing this possible confounding event in our analysis. It is clear that rural residence increases exposure to *P. vivax,* but it is not clear which behaviors from mothers living in rural communities contribute to increased inflammation and poor pregnancy outcomes. We have now applied a multivariate analysis considering the suggested confounding variables (maternal age, gravidity, occupation, residence, and educational level). Only first trimester infections were found to be statistically significantly associated with adverse pregnancy outcomes. Therefore, we have now re-analyzed the whole dataset and associated findings since the new approach changed the manuscript scope and hypothesis. Although the new model’s performance did not have a considerable improvement compared with the former model, the new set of results generated still suggest increased association between infections and some of the poor outcomes when comparing with the previous analysis. In fact, it evidences that poor outcomes are associated with *P. vivax* first trimester infections (without being affected by the gravidity status of pregnant women).

**Results**

*3) Univariate analyses are used throughout. A multivariate approach is strongly recommended (see preceding section).*

R: As suggested by the reviewer, we have now conducted multivariate analysis throughout the manuscript adjusting the analysis for the same confounding effects previously described (maternal age, gravidity, educational level, occupation, and residence). Accordingly, we have now re-analyzed the whole dataset and associated findings since the new approach changed the manuscript scope and hypothesis.

**Conclusions**

*4) Until the data have been re-analyzed with inclusion of confounding factors, I cannot confidently evaluate the appropriateness of the authors’ interpretations of their data, or the conclusions they draw.*

R: We are replying the reviewer’s comment in the previous questions. We hope that the newly re-analyzed dataset generated more clear results.

**Reviewer #2**

Objectives of this study are well stated. Many important details of the methods, especially of sampling from the participants, are omitted. I expect that previous publications provide this information, but readers of this article should not have to seek out those papers in order to understand the study.

**Methods**

*1) It would be helpful to know (briefly) how the women in this study were recruited – before or during pregnancy, at home or clinic or hospital, and how followed – monthly, active or passive follow-up? What exactly defines a woman ‘with P vivax?’ – detected infection at any time in the pregnancy, or at parturition, or in the placenta, or any of these? In general we need to be told when samples were taken – some can obviously be taken only at or around parturition, but others could be taken at various times during pregnancy: e.g. at what point in the study were blood samples taken from women studied for total IgG antibodies against Plasmodium vivax Merozoite Surface Protein 1 (PvMSP1-19)? (Was evidence being sought for P vivax infection before pregnancy had started, or at some stage in the current pregnancy before enrolment?)*

R: We acknowledge this comment; we have now added the information below into the Methods to clarify this point better:

“Briefly, 600 pregnant women were recruited during their first antenatal care (ANC) visit. These women were followed until delivery, between January 2013 and April 2015. Data was collected on socioeconomic, clinical, and obstetric variables at the time of recruitment, together with peripheral blood samples. In parallel, thick and thin peripheral blood smears were performed to screen for malaria parasites by microscopy, which were later confirmed by PCR. Each pregnant woman was followed by a trained nurse, which involved at least two domiciliary visits, at the second and third trimester, to monitor their clinical state and collect a peripheral blood sample, in addition to the usual prenatal care visit to the health care services. An additional blood sample was collected in each malaria episode during pregnancy. At the time of delivery, clinical data were collected from mother and newborn and performed a placental biopsy and blood sampling.”

All pregnant women enrolled were screened for malaria at enrolment, during pregnancy, and at delivery by microscopy blood smear, and confirmed by molecular diagnostic PCR. Only after this confirmation by PCR were pregnant women classified as non-infected, infected by Pv, Pf or mixed infection.”

In this study, samples were collected as follows:

- At recruitment (first trimester) – peripheral blood (5 ml)
- First domiciliary visit (second trimester) – peripheral blood (5 ml)
- Second domiciliary visit (third trimester) – peripheral blood (5 ml)
- At delivery – peripheral blood (5 ml); placental biopsy (2x2 cm3 in formalin and 2x1 cm3 in RNA stabilizer); placental blood (5 ml); and umbilical cord blood.

For the study of total IgG antibodies against PvMSP1-19, samples collected at the first timepoint (recruitment) were used, because we were looking for evidence of *P. vivax* infections before pregnancy had started. We also removed from the analysis infected pregnant women who were recruited with more than one malaria during current pregnancy to increase the probability of targeting antibodies acquired after malaria episodes that occurred before pregnancy. We have now added this information in Methods section.

*2) Were tests for Pv taken at first recruitment irrespective of symptoms (e.g. fever); were women asked to attend for any fever; were blood films taken routinely at scheduled visits to the clinic, or only if there were symptoms? Did the ‘endemic surveillance team’ actively seek women with fever, or women in pregnancy, to enrol into the study?*

R: Yes, all pregnant women were tested at recruitment, during domiciliary visits and at delivery. In addition, whenever they had any symptoms during pregnancy (e.g. fever, indisposition, headache etc.), sample collection was also performed for testing.

Pregnant women participated in the study on a voluntary basis, generally occurred in two ways: non-infected pregnant women were invited to participate in the study during their first visit to the antenatal care clinic; and infected pregnant women were invited to participate in their first diagnosis of malaria in the endemic service, which then occurred passively.

*3) Page 11. When assessed by microscopy, were placental samples ‘blinded’ as to P vivax infection in the mother?*

R: Thanks for pointing this out. We have now added the following sentence: “*All measurements were performed by two investigators blinded to group and outcome. Cases that proved to be contradictory between observers were re-evaluated until consensus was reached.*” in Methods section.

**Results**

*4) Many data are presented in detail in this paper, both in text and Figures, so that the important findings are difficult to identify. It would be useful to decide a hierarchy of importance of findings and make sure that these are conveyed prominently.*

R: We thank the reviewer for this suggestion. Since we restructured the manuscript upon suggestions from Reviewer 1 regarding multivariate analysis, we had the opportunity to better define the most important results and distribute them accordingly. As such, we eliminated some graphs from the main manuscript (former Figure 2, 6 and 7) and data analysis from cytokines and angiogenic factors are only presented as supplementary data (new S3 and S4 Table).

*5) P14 line 290: “All p values were 2-sided, and interpreted at a significance level of 0.05.” Mention should be made that there are very large numbers of statistical tests in the Tables and figures – will any adjustment for multiple testing be made to the interpretations of ‘significance’?*

R: In this manuscript, we did not consider using multiple testing corrections for the interpretation of significance. Multiple testing adjustment methods such as False Discovery Rates (FDR) are known to diminish the probability of committing type I errors when a considerable number of hypotheses are tested within a specific set of data obtained from a given experiment (e. g. omics analysis and microarrays). However, it also increases the chances of committing type II errors, masking possible important findings within a given set of data (Feise 2002). In our case, we do not believe that the number of hypotheses tested justifies applying adjustment such as FDR, which usage could be applied when thousands of tests are performed (e. g. omics and arrays) (Higdon et al. 2008). In fact, since microarrays’ and omics’ targets are predetermined, the number of tested hypotheses is generally fixed, as opposed to our dataset from which variables could be omitted, impacting the p values and possibly increasing the probability of false discoveries (Feise 2002).

*6) In Fig 4 please clarify why denominators in column 2 don’t add up to the same total on each line.*

R: This is because not all measurements were verified in all newborns (following the pattern: immediately after delivery or maximum within a 24-hour period). Example: of the total of 329 babies, only 319 had their weight checked immediately after birth; only 313 had their head circumference measured, etc.

**Conclusions**

*7) There is frequent reference (or implication) in this paper to a finding being the cause of some later event or difference. For example: p32 lines 557-558: “we have observed an important impact of P. vivax infection on fetal growth…”. In fact association has been observed, but you have not demonstrated a causal link (implied by ‘impact’). The above, for example, would be better written as ‘we have observed* *a significant association between P vivax infection and impaired fetal growth…’. Several implications or assumptions of causality recur throughout the paper and would be better reworded appropriately (including in the last sentence of Discussion).*

R: We have now removed “causal” language throughout the text.

**Editorial and Data Presentation Modifications?**

*8) One woman in her first pregnancy is a primigravida, more than one are primigravidae. Please correct ‘primigravida’ (and ‘multigravida’) throughout the paper, including in Tables and Figures, where they should be plural nouns.*

R: Since we restructured the manuscript upon Reviewer 1 suggestions regarding multivariate analysis, the scope of our manuscript changed to infection trimester rather than gravidity. Nevertheless, we have corrected the text, tables and figures whenever necessary.

*9) p.21 line 348: 'Pv-infected women experienced more adverse pregnancy outcomes'…Several references are made to ‘more’ or ‘higher’ – when referring to differences between groups; in these situations please add ‘on average’ or ‘mean’ or ‘median’ before the comparative word, and make sure you indicate with whom or against what the comparison is being made. For example P32 line 571 “we observed that primigravida who presented infection in the second trimester had higher parasitemia.” Higher than whose? Than you would expect? Or than multigravidae with 2nd trimester infections? Or than primigravidae with infections in the first trimester? Etc… Please look out for comparatives without a comparator, and clarify accordingly.*

R: We appreciate this comment, and the manuscript was revised to clarify all comparisons whenever possible.

*10) Throughout the paper, especially in the Discussion, redundant words are commonly used to start sentences. Try omitting these and see if it makes any difference to the clarity of the text (I don’t think it does): examples: ‘Additionally’, ‘Finally’ (best avoided because you can never say the final word on any subject), ‘Not surprisingly’, ‘Further’, ‘In addition’, ‘Remarkably’, ‘Furthermore’, ‘Moreover’, ‘Controversially’, ‘Thus’.*

R: We acknowledge this comment and have now removed those terms from the manuscript.

**Summary and General Comments**

*11) The importance of this study is diminished by the profusion of material - more brevity in the introduction and discussion, and a clarification of findings of importance, would make it more accessible to readers. At present the paper would do better for a thesis-Chapter than a journal publication.*

R: We agree that the Introduction and Discussion are extensive; however, we have now tried to make it concise within the constraints of the PLOS NTD guidelines.

**Reviewer #3**

The authors present the results of a longitudinal study of *P. vivax* malaria in a population of pregnant women in Brazil. This study focuses exclusively on women with P. vivax infection identified through passive case detection and at delivery compared to non-infected pregnant women. Extensive data are provided on the clinical outcomes, placental histology, newborn characteristics and analyses of various biomarkers. The study reveals that *P. vivax* infection in pregnancy, particularly during the first trimester, can be detrimental to the health of the newborn, prompting re-consideration of the severity of infections with this species in pregnancy. While the study provides very important data on *P. vivax* MiP that will contribute significantly to the field, there are a number of limitations and these should be discussed.

**Methods**

*1) Details on the timing of recruitment and follow up in the study are not adequately described. At what gestational age were women recruited into the study? How many malaria episodes occurred during pregnancy?*

R: This issue was also raised by another reviewer. We have now incorporated more information about the follow-up in the Methods section to better clarify this point.

“Briefly, 600 pregnant women were recruited during their first antenatal care (ANC) visit. These women were followed until delivery, between January 2013 and April 2015. Data was collected on socioeconomic, clinical, and obstetric variables at the time of recruitment, together with peripheral blood samples. In parallel, thick and thin peripheral blood smears were performed to screen for malaria parasites by microscopy, which were later confirmed by PCR. Each pregnant woman was followed by a trained nurse, which involved at least two domiciliary visits, at the second and third trimester, to monitor their clinical state and collect a peripheral blood sample, in addition to the usual prenatal care visit to the health care services. An additional blood sample was collected in each malaria episode during pregnancy. At the time of delivery, clinical data were collected from mother and newborn and performed a placental biopsy and blood sampling.”

All the women who presented malaria during pregnancy were treated with antimalarial drugs under medical prescription, according to the Brazilian Ministry of Health (MoH) guidelines.

The number of malaria episodes ranged from 1 to 5 infections during pregnancy.

*2) Given that the women were not followed during pregnancy unless they were symptomatic, how can the authors rule out that they did not experience a P. falciparum infection at some point in pregnancy that was asymptomatic?*

R: All pregnant women (non-infected and infected) were followed up during pregnancy, as described above. Therefore, we make sure that there was no undetected asymptomatic infection, as blood samples were collected at different time points during pregnancy, in addition to the monitoring performed by the local health system. It is worth mention, as stated earlier, that all blood samples collected were tested by PCR for diagnostic confirmation.

*3) Table 1 should include statistical analyses to compare across the different groups.*

R: As requested by the reviewer, we have now included statistical analysis for the different comparisons in Table 1.

*4) How were previous P. vivax infections in the current or prior pregnancies confirmed? (Line 312)*

R: Information about previous malaria - malaria before current gestation (in life) and malaria in previous pregnancy (in other previous pregnancies) - was reported verbally by pregnant women. This information was collected through a questionnaire. However, so that we could use this data more precisely, we used the study of total IgG against the protein PvMSP1-19, which is one of the most studied markers of infection in *P. vivax* malaria.

**Results**

*5) In Fig 8, why would newborns have longer length in the uninfected group depending on their PvMSP1 Ab status?*

R: Since we restructured the manuscript upon Reviewer 1 suggestions regarding multivariate analysis, this length difference between the groups was lost.

*6) It seems possible that the PvMSP1 antibodies at enrolment could be due to a recent infection in that pregnancy prior to enrolment, rather than an exposure prior to pregnancy. Consideration of the timing of infection and timing of enrolment are important to interpret these serology data.*

R: We thank the reviewer for this pertinent question, which made us realize some abnormalities in the analysis that could have led to erroneous conclusions. In fact, we used plasma collected from the first timepoint (recruitment); yet, for some infected pregnant women, the recruitment occurred after they have already experienced an episode of malaria during that pregnancy, being recruited during their second or even during their third episode of malaria. Accordingly, we have re-analyzed data after excluding data from pregnant women who had their recruitment and first blood collection after they have already experienced malaria during that current pregnancy. This will now remove the bias introduced by recent infections prior to recruitment from this analysis, where we kept only data collected from infected women who had their first collection timepoint in their first infection during pregnancy. In this case, we believe that the measured IgG antibodies were not related to current infection as antibodies should only be generated approximately within a week after exposure. Since pregnant women in this study rapidly sought medical aid after developing malaria, we truly believe that the measured antibodies were developed against infections contracted before their current pregnancy, despite the increased levels of IgG against P. vivax MSP1 observed in the infected group in comparison with their non-infected counterparts. This also correlates with their verbal consent of previous malaria episodes; from those 231 that reported previous malaria episodes (92 non-infected plus 139 infected) (Table 2), 77% had IgG against P. vivax MSP1 (data not shown). With this, we have now reinterpreted our findings in the manuscript.

**Conclusions**

*7) The statement on lines 73-75 is not entirely accurate. The study by Bardaji et al (2017), for example, explored some of these interactions between Pv infection in pregnancy and birth outcomes.*

R: We acknowledge this comment and have now modified this statement to better clarify this point. So far there is no study that assesses all these points together. Important contributions to this field were done by François Nosten and Rose McGready about 20 years ago in South East Asia and more recently by Alfredo Mayor, Kerryn Moore, AnaClara Pincelli, Azucena Bardají and others, which also conducted studies in the Americas and Thailand; yet, most of these manuscripts focus solely in addressing either placental histopathology or poor pregnancy outcomes. In fact, some manuscripts even focus their efforts on *P. falciparum* malaria in pregnancy, occasionally studying *P. vivax* infections during pregnancy in parallel. Hence, we are stating that this manuscript is one of the few studies, if not the only, that combines data from pregnancy outcomes, placental histopathology, inflammatory mediators, angiogenic factors, and serology.

*8) The statement on lines 83-85 should be revised since these associations were observed in specific groups, not generally between infected vs non-infected women, except for preterm birth as shown in Fig 2. The statement should also reflect the data in Figures 3 and 4.*

R: Since we restructured the manuscript upon Reviewer 1 suggestions regarding multivariate analysis, the scope of our manuscript changed to infection trimester rather than gravidity. We hope we have addressed this issue in the new version of the manuscript.

*9) Line 86. Placental malaria is defined by the presence of parasites or hemozoin in the placenta, not by these histological changes in the placenta. This sentence should be revised accordingly. Only 8 women in the study had placental malaria and the results should be interpreted with some caution.*

R: We accept this suggestion and have now changed it in the manuscript.

10) I don’t agree with the statement on line 351. The difference is only significant for infections in the second trimester. The results seem to be skewed by the high error bars.

R: Since we restructured the manuscript upon Reviewer 1 suggestions regarding multivariate analysis, the scope of our manuscript changed to infection trimester rather than gravidity. Accordingly, Figure 2 was removed from the manuscript.

*11) The strong conclusions from the serology data for PvMSP1-19 are premature. The authors state that they used this marker as a surrogate of past exposure to P. vivax and yet they conclude that these antibodies are associated with protection. Was there a correlation between PvMSP1 Abs and placental outcomes or clinical outcomes? The discussion of pre-existing immunity should be removed or revised substantially.*

R: Since we restructured the manuscript upon Reviewer 1 suggestions regarding multivariate analysis, the results and conclusions have changed. Additionally, as the reviewer suggested, we have now measured possible associations between anti-PvMSP1 IgG and pregnancy outcomes with multivariate analysis (new Fig 5B). As such, no association was found between IgG titers and improved outcomes in the context of *P. vivax* malaria in pregnancy. We have now discussed these findings in the new version of the manuscript.

**Editorial and Data Presentation Modifications?**

*12) Minor comments:*

- Line 159. Please state EIR or some other measure of malaria transmission in the study area.

R: We have now added the Annual Parasite Incidence (API) information in the Methods section.

- Specify source of PvMSP1-19 protein used for ELISAs and concentration coated on the plate.

R: We have now added this information in the Methods section: “Enzyme-linked immunosorbent assay (ELISA) was performed using high-binding 96-well microplates (Costar, Cambridge, MA) coated with Glutathione S-transferase (GST)-PvMSP119 (31.25 ng/well), produced as described elsewhere (de Oliveira et al. 1999, Bastos et al. 2007). In parallel, GST was used as a control for non-specific binding. The optimal concentrations were determined by plasma serial titration of positive and negative controls.” Since the protein was obtained by one of the coauthors (Gerhard Wunderlich) and the cloning and synthesis are described elsewhere, we did not detail this information in the manuscript.

- Table 3. Include the units for the parasitemia

R: We have now inserted the units in the footnotes of the table.

*13) Minor grammatical errors, as follows:*

- Line 61, delete ‘occurring’

R: We have now corrected the text in the new version of the manuscript.

- Line 122, rephrase ‘it is detected’

R: This was rephrased.

- Line 150, change the term ‘benignity paradigm’

R: We have now corrected the text in the new version of the manuscript.

- Line 183, should be ‘first’

R: This was corrected accordingly.

- Line 240 should be ‘10th percentile’

R: We have now corrected the text in the new version of the manuscript.

- Line 308 should be ‘pregnant women’

R: We have now corrected the text.

- Line 316, change ‘exert’ to ‘may be detrimental’

R: This was changed accordingly.

- Line 377, remove ‘the’

R: We have now corrected the text in the new version of the manuscript.

- Line 379, ‘in’ should be ‘on’

R: We corrected this.

- Line 385, Change the word ‘patent’, perhaps to ‘apparent’ or another term

R: We changed the word accordingly.

- Line 387, change ‘multigravidity status’ to ‘in multigravida’

R: We have now corrected this throughout the text.

- Line 430, should be ‘alterations in several’

R: We have corrected this.

- Line 457 should be ‘cytokine’

R: This was corrected.

- Line 460, remove period before bracket

R: Period removed.

- Line 461, change ‘of’ to ‘in’

R: “Of” was changed to “in” at this point.

- Line 484, please change the term ‘tenuous’ – the meaning here is not clear

R: The term was changed.

- Line 489, change ‘heighten’ to ‘greater’

R: This was changed accordingly.

- Line 509, change to samples collected at enrollment

R: This was changed accordingly.

- Line 540 is repetitive with line 535

R: The repetition was removed.

- Line 560, ‘of’ should be ‘on’

R: “Of” was changed to “on”.

- Line 561, add a comma after infections

R: Comma added.

- Line 579, remove ‘the existence of’, line 580 ‘supports’

R: We changed this accordingly.

- Line 602, change ‘though’ to ‘however’

R: We changed this accordingly.

- Line 627 should be ‘protection’

R: We inserted “protection”
